# Bioenzyme-based nanomedicines for enhanced cancer therapy

**DOI:** 10.1186/s40580-022-00297-8

**Published:** 2022-02-04

**Authors:** Mengbin Ding, Yijing Zhang, Jingchao Li, Kanyi Pu

**Affiliations:** 1 Shanghai Engineering Research Center of Nano-Biomaterials and Regenerative Medicine, College of Chemistry, Chemical Engineering and Biotechnology, Donghua University, Shanghai, 201620 China; 2 School of Chemical and Biomedical Engineering, Nanyang Technological University, 70 Nanyang Drive, Singapore, 637457 Singapore

**Keywords:** Enzyme, Nanomedicine, Cancer therapy, Tumor microenvironment, Drug delivery system

## Abstract

Bioenzymes that catalyze reactions within living systems show a great promise for cancer therapy, particularly when they are integrated with nanoparticles to improve their accumulation into tumor sites. Nanomedicines can deliver toxic bioenzymes into cancer cells to directly cause their death for cancer treatment. By modulating the tumor microenvironment, such as pH, glucose concentration, hypoxia, redox levels and heat shock protein expression, bioenzyme-based nanomedicines play crucial roles in improving the therapeutic efficacy of treatments. Moreover, bioenzyme-mediated degradation of the major components in tumor extracellular matrix greatly increases the penetration and retention of nanoparticles in deep tumors and infiltration of immune cells into tumor tissues, thus enhancing the efficacies of chemotherapy, phototherapy and immunotherapy. In this review, we summarize the recent progresses of bioenzyme-based nanomedicines for enhanced cancer therapy. The design and working mechanisms of the bioenzyme-based nanomedicines to achieve enhanced chemotherapy, photothermal therapy, photodynamic therapy, chemodynamic therapy, radiotherapy and immunotherapy are introduced in detail. At the end of this review, a conclusion and current challenges and perspectives in this field are given.

## Introduction

Bioenzymes that catalyze the conversion of substrate molecules into products in living systems have been used as a class of alternative therapeutic agents for treatments of cancer [1–4]. Compared to traditional small-molecular drugs, enzymes show the advantages of high bioactivity, good specificity, negligible multidrug resistance and relatively low adverse effects [5–7]. In preclinical studies, enzymes can directly induce the death of cancer cells [8], or improve the efficacy of chemotherapy [9]. However, enzymes often have the problems of low stability, short blood circulation property, and poor membrane permeability, which greatly hampers their bioavailability and applications for in vivo cancer therapy [10–12]. Although frequent injection of enzymes is oftentimes mandatory to improve the therapeutic benefits, which will result in high costs and some adverse effects [13–15]. Therefore, development of suitable approaches for enzyme delivery is an urgent need.

Nanomedicines have been extensively used to improve the potencies of cancer therapy [16–19]. On the one hand, nanoparticles themselves can serve as therapeutic agents to treat tumors via generating heat or highly toxic reactive oxygen species (ROS) upon external stimuli [20–22], or reactions with endogenous chemical energy in tumors [23–25]. On the other hand, nanoparticles can integrate small-molecular drugs [26–29], genes [30], and enzymes [31] to improve their pharmacokinetics, and allow their accumulation and release in tumor sites to exert functions. Using nanoparticles to encapsulate enzymes is able to improve their stability and circulation for high bioavailability and effective accumulation in tumors [32–34]. In addition, controlled release of enzymes from nanoparticle-based delivery systems allows targeting to specific sites, leading to improved efficacy and reduced side effects [35]. In view of these advantages, enzyme-based nanomedicines have been developed for cancer therapy. In addition to direct killing of cancer cells, enzyme-based nanomedicines can modulate tumor microenvironment to enhance anticancer efficacy.

**Fig. 1:**
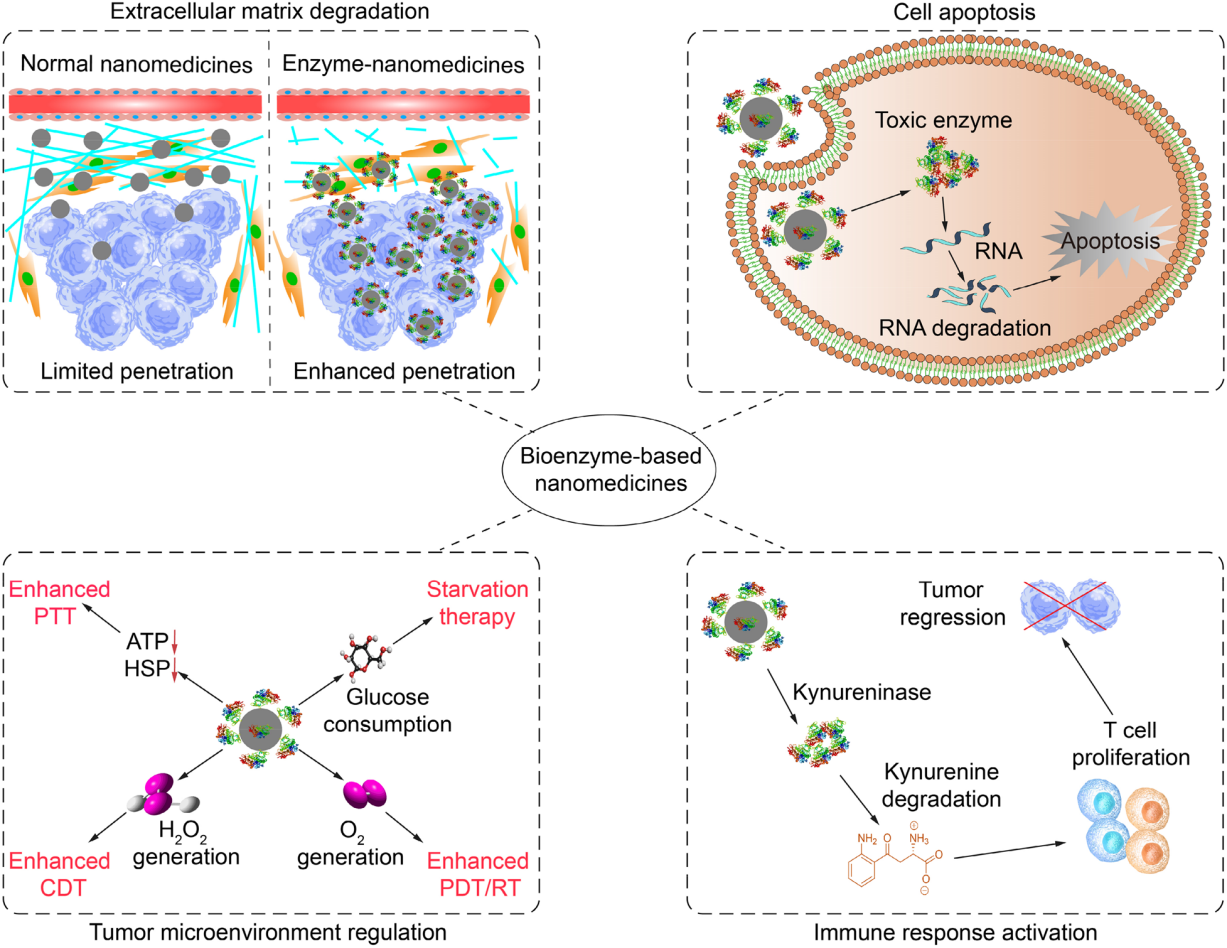
Summary of bioenzyme-based nanomedicines for enhanced cancer therapy via degradation of extracellular matrix, induction of cell apoptosis, regulation of tumor microenvironment, and activation of immune responses

In this review, we summarize recent advances of bioenzyme-based nanomedicines for enhanced cancer therapy. The construction and work mechanisms of bioenzyme-based nanomedicines to achieve enhanced chemotherapy, photothermal therapy (PTT), photodynamic therapy (PDT), chemodynamic therapy (CDT), radiotherapy (RT), and immunotherapy of solid tumors are introduced (Fig. 1). Then, a brief summary and outlook are given along with the discussion of the existing challenges and perspectives in this field.

## Bioenzyme-based nanomedicines for chemotherapy

Some cytotoxic enzymes can directly induce cell death and thus can be used for cancer therapy. Nanomedicines have been utilized to control the release of enzymes, achieving improved therapeutic efficacy and reduced side effects. Chen’s group reported a hypoxia-sensitive supramolecular nanogel for the cytosolic delivery of ribonuclease A (RNase A) to treat breast cancer [36]. The supramolecular RNase A-loaded nanogels were formed through a self-assembly of the host–guest interaction between azobenzene (Azo) and β-cyclodextrin (β-CD) grafted onto poly (L-glutamic acid)-graft-poly (ethylene glycol) methyl ether (PLG-g-mPEG), followed by loading of RNase A inside nanogels under mild aqueous conditions though a direct mixing method. In addition, a nano-formulation of vascular disrupting agent PLG-g-mPEG/combretastatin A4 (nano-CA4) was synthesized to further enhance the hypoxia status of tumors. After intravenous injection, RNase A-loaded nanogels and nano-CA4 were internalized by cancer cells via multiple pathways including macropinocytosis and clathrin-dependent endocytosis. With the presence of nitroreductase (NTR) in most hypoxic cancer cells, the cross-linking point between Azo and β-CD was destroyed due to the conformation transition of Azo, achieving the hypoxia-sensitive release of cargo from the nanogels. RNase A nanogels with significantly prolonged stability in the circulation exhibited an enhanced antitumor efficacy compared to free RNase A in 4T1 tumor mouse model without causing obvious systemic toxicity. When combined with nano-CA4, the hypoxic environment of the tumors accelerated the release of RNase A, leading to inhibition of tumor growth with a tumor suppression rate up to 91.7%.

In another study, Leong’s group constructed a bioinspired diselenide-bridged mesoporous silica nanoparticle (MSN) with oxidative and redox dual-responsive delivery of RNase A for cancer therapy [37]. The diselenide-bond-containing organosilica moieties were integrated into the mesoporous silica framework to fabricate large-pore MSNs, which were used to load RNase A and surface coating with cancer cell membrane. The presence of cancer cell membrane coating realized a homologous targeting and shielding from the immune system, which would be beneficial to accumulation of nanoparticles into tumors and achieved efficient cellular uptake via lysosome-dependent pathway. Both the oxidative and reducing conditions due to the presence of ROS and glutathione (GSH) in cancer cells induced the interruption of diselenide bond in nanoparticles to release RNase A for cancer cell killing. As a result, the nanoparticles exhibited significant anti-cancer effect in HeLa tumor-bearing nude mice.

Co-delivery of enzymes and drugs can also achieve enhanced therapeutic efficacy. Ma’s group recently developed a single delivery nanosystem to release cis-platinum pro-drugs (DSP) and RNase A for cancer therapy [38]. The nanosystem was synthesized via encapsulating quadrivalent DSP and RNAse A into large-pore mesoporous silica-coated upconversion nanoparticles. RNAse A can be used for ribonucleic acid (RNA)-targeted cancer therapy because it degrades messenger RNA and transfer RNA to inhibit the expressions of proteins. After the nanoparticles were internalized by tumor cells, DSP and RNAse A were released from the pores of nanoparticles, and RNAse A degraded RNA, and activated DSP caused DNA damage, achieving the combination of chemotherapy and enzyme therapy. Thus, the synergetic action of chemotherapy and enzyme therapy showed a better therapeutic effect in vivo in comparison with chemotherapy or enzyme therapy alone. Besides, the nanoparticles had conversion luminescence and magnetic resonance dual-mode bioimaging properties, which allowed for precise tumor treatment.

**Fig. 2:**
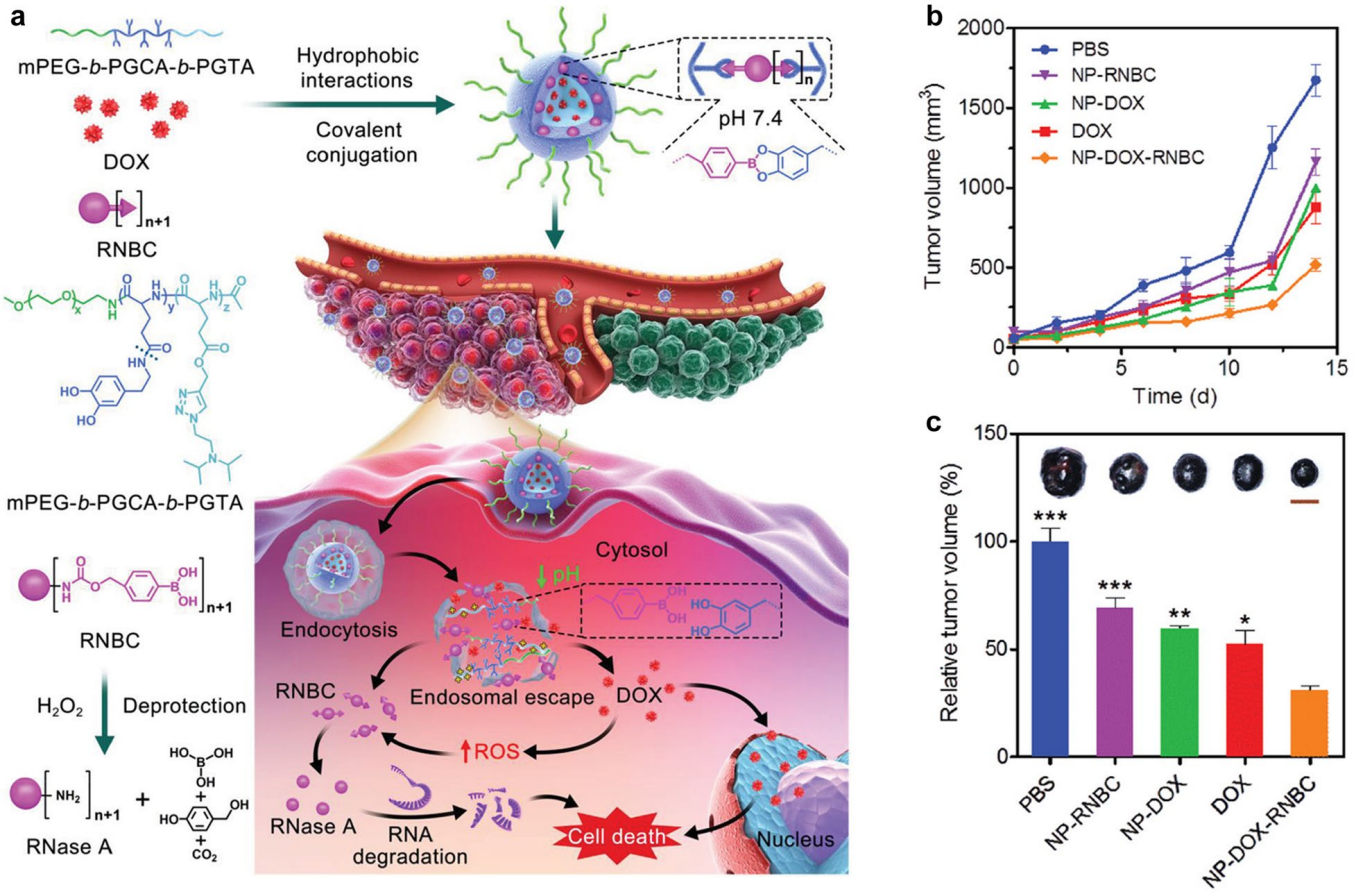
**a** Schematic illustration of the intracellular co-delivery of RNase A and DOX using a multistage cooperative drug delivery nanoplatform formed by mPEG-b-PGCA-b-PGTA for combination cancer therapy. **b** Tumor volume changes of B16F10-tumor-bearing mice after intravenous injection of PBS, NP-RNBC, NP-DOX, DOX, and NP-DOX-RNBC on day 0, 3, 6, and 9. **c** Relative tumor volume of the mice treated as described in (**b**) on day 14. Inset: Representative photographs of the excised tumors from the mice treated as described in (**b**) on day 14. Reproduced with permission from [39]. Copyright 2020, Wiley–VCH

In another study, Chen’s group developed a multistage cooperative drug delivery nanoplatform to overcome integration barriers caused by different physicochemical properties for synergizing single protein therapeutics and small-molecule chemotherapeutics [39]. An amphiphilic triblock copolymer, mPEG-b-PGCA-b-PGTA was synthesized to encapsulate doxorubicin (DOX) through a hydrophobic interaction with the unprotonated PGTA block, and RNase A modified with a nitrophenyl tetramethyl-dioxaborolanyl benzyl carbamate group (RNBC) was then attached to the PGCA block to crosslink the nanoparticles through phenylboronic acid-catechol interactions, resulting in the formation of such nanoplatforms (Fig. 2a). Upon accumulation in tumor sites, PGTA block changed into hydrophilic via protonation of tertiary-amine side groups at acidic pH, resulting in a pH-responsive release of DOX. In acidic endolysosomes, RNBC was released due to acidic pH-triggered cleavage of phenylboronic acid-catechol linkages and subsequent endolysosomal escape induced by the “proton sponge” effect of tertiary-amine-containing PGTA block. DOX caused cell apoptosis and also increased intracellular ROS levels. The increased intracellular ROS levels further facilitated the conversion of RNBC into bioactive RNase A via ROS-triggered deprotection, resulting in a synergistically enhanced anticancer effect of RNase A and DOX (Fig. 2b and c). Such a treatment greatly inhibited the growth of B16F10 tumors in mice and improved the mouse survival rates.

In addition to direct cytotoxicity to cancer cells, enzymes can be utilized to enhance the efficacy of drugs. Chen’s group constructed an organosilica-based mesoporous bilirubin nanoparticle to achieve the combination of starvation therapy and chemotherapy mediated by glucose oxidase (Gox) and a prodrug tirapazamine (TPZ), respectively [40]. Gox was covalently conjugated onto the surface of nanoparticles through dehydration condensation between the Si–OH groups of organosilica and Gox-silane, and the positively charged TPZ was loaded into the cavity of negatively charged nanoparticles via electrostatic adsorption, followed by surface modification of PEG onto nanoparticles to increase biocompatibility for in vivo experiments. The nanoparticles degraded the shell through the low pH value in the tumors, and the H_2_O_2_ produced by Gox further accelerated the release of TPZ. Gox competed with tumor cells for glucose to achieve starvation therapy. TPZ was activated into active form through Gox-mediated consumption of O_2_ inside the tumor for chemotherapy. Thus, such nanoparticle-mediated glucose and O_2_ depletion could starve tumors and simultaneously increase the hypoxia-activated TPZ toxicity to significantly improve deoxygenation-driven synergistic starvation-enhanced chemotherapeutic effect, greatly inhibiting the growth of U87MG tumors.

Enzymes can also increase the efficacy of chemotherapy via decreasing drug efflux. Cai’s group reported a core–shell therapeutic nanoparticle with stimulus responsive and sequential drug release for enhanced cancer chemotherapy [41]. In such a nanoplatform, DOX and Gox was loaded in the core of mesoporous polydopamine and shell of zeolite imidazolate frameworks-8, respectively. The high DOX loading capacity of nanoparticles might be attributed to the mesoporous structure, π-π stacking and the hydrophobic interaction between mesoporous polydopamine and DOX. Due to the acidic in tumor microenvironment, the shell was degraded to release Gox, which consumed intracellular glucose to deprive adenosine triphosphate (ATP) to inhibit the function of ATP-dependent P-glycoprotein (P-gp) transporter for decreasing drug efflux. Furthermore, the produced gluconic acid further accelerated the degradation of shell to release more Gox. Subsequently, DOX inside the pores of mesoporous polydopamine was released and less be pumped out benefiting from the above effects, which enhanced the accumulation of DOX to reserve multidrug resistance of cancer cells. In addition, the produced H_2_O_2_ through the catalytic action of the released Gox could induce oxidative damage to cancer cells and accelerate their apoptosis. As such, the nanoparticle-mediated therapy showed a super antitumor effect in inhibiting the growth of MCF-7 tumors.

Through increasing the tumor penetration of drug-based nanoparticles, enzymes can also achieve enhanced chemotherapy of cancer. Wu’s group developed a tumor microenvironment-responsive co-modified nanocarrier with loading of paclitaxel (PTX) and Gox for amplified chemotherapy of breast cancer [42]. A cell-penetrating peptide (R8) was attached to the surface of nanocarriers to allow their strong deep penetrability and endosomal escape. In addition, a long chain biotin-PEG was introduced to the nanocarriers via an acid-sensitive hydrazone bond to shield the positive charges of R8 peptide. PTX and Gox was then loaded into tumor-targeted nanocarriers. Biotin had a specifically bind to the sodium-dependent multivitamin transporter to achieve tumor targeting. At acidic tumor environment, the nanocarriers underwent slow hydrolysis of the hydrazone bond with consequent exposure of R8 peptides. The Gox-mediated oxidation of glucose increased the acidity, which could further accelerate the breakage of hydrazone bond, and thereby promoted the deep penetration of nanoparticles to amplify the therapeutic efficacy. Such a synergistic action of Gox-mediated starvation therapy and PTX-based chemotherapy enhanced the antitumor effect, greatly inhibiting the growth of 4T1 tumors.

To promote the accumulation of DOX at the tumor sites for effective and accurate tumor treatment, Chen’s group constructed a hyaluronidase (HAase)-loaded multi-responsive nanoplatform to stepwise co-deliver an enzyme and prodrug [43]. Such a nanoplatform consisted of a polyester-hyaluronic acid-DOX (PE-HA-DOX) prodrug as the corona, physiologically biodegradable silica containing disulfide bonds as the shell, and inside encapsulated HAase as the core. HA-DOX was non-toxic due to its large molecule weight that mismatched with the size of nuclear pores. After intravenous injection, the nanoplatforms showed an increased accumulation into tumors through CD44-HA ligand-receptor-mediated targeting pathway. Silica shells with disulfide bonds and polyester were broken in the tumor microenvironment due to the high levels of esterase and GSH, leading to release of HA-DOX and HAase in a stepwise manner. HAase catalyzed the decomposition of HA-DOX to form highly toxic dissociative DOX for inducing apoptosis and death of tumor cells. The SCC tumors of nude mice were remarkably inhibited with only a little growth after the nanoplatform-mediated treatment.

**Fig. 3:**
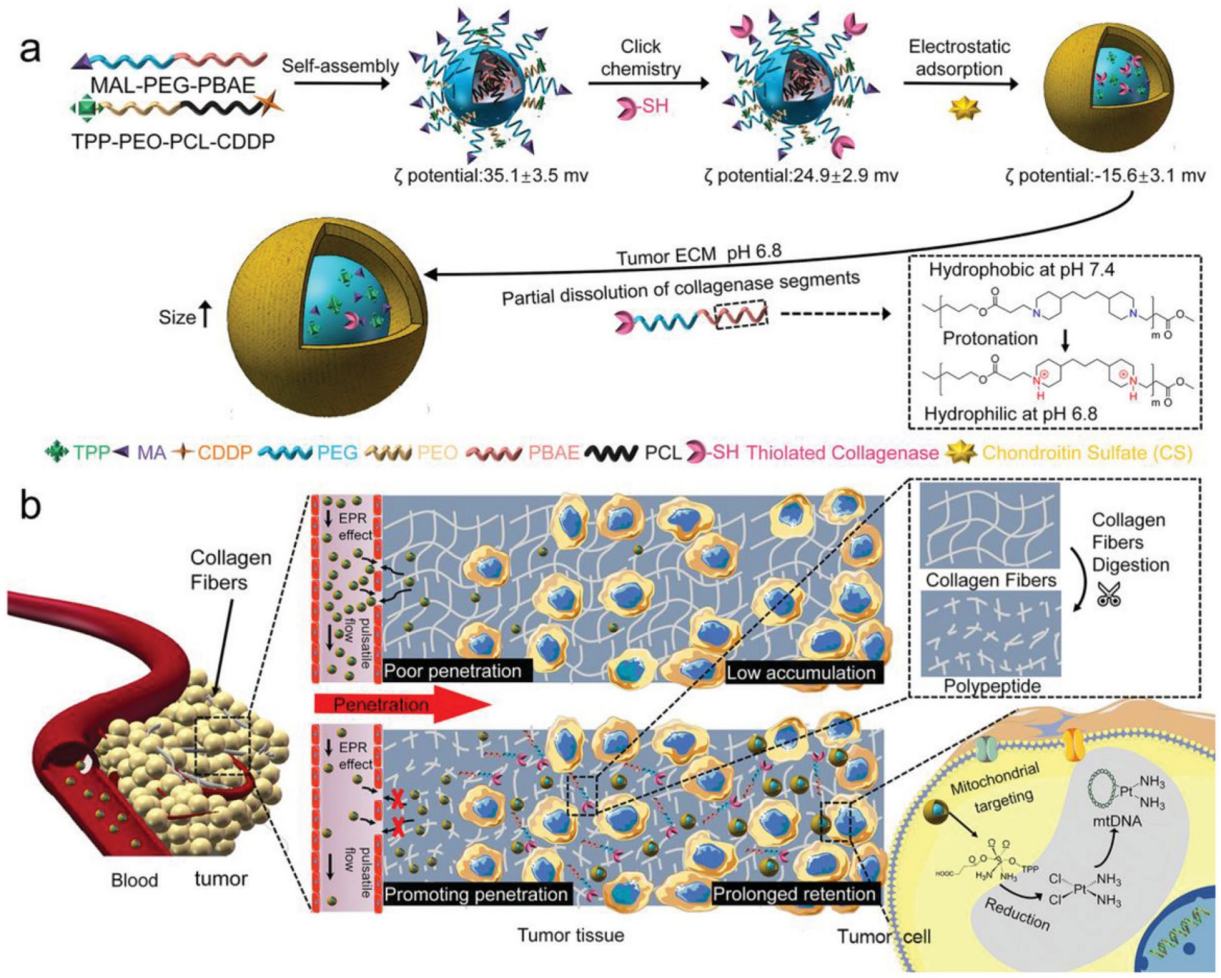
**a** Schematic illustration of the fabrication of collagenase-based nanoscavengers and their size increase and dissociation of the collagenase containing components in response to the acidic pH. **b** Schematic illustration of the increased penetration and retention of nanoparticles in deep tumor tissues via the combined action of collagenase digestion of collagen fibers and particle size increase, and the destruction of mitochondrial DNA via mitochondria-specific targeting and release of cisplatin drugs into mitochondria. Reproduced with permission from [45]. Copyright 2020, Wiley–VCH

Dense extracellular matrix (ECM) in tumor microenvironment often leads to limited penetration and inadequate retention of nanoparticles in deep tumors, greatly hampering their tumor accumulation capability for cancer therapy [44]. Bioenzymes that can degrade the major components of tumor ECM have been used to address these barriers for enhanced cancer chemotherapy. As reported by Zhou’s group, a size-changeable collagenase-modified polymer nanoscavenger was developed to enhance the penetration and retention of nanomedicine in deep tumor tissue for cancer therapy [45]. A micelle was formed via a self-assembly of maleimide-terminated poly(ethylene glycol)-block-poly(β-amino ester) (MAL-PEG-PBAE) and succinic anhydride-modified cisplatin-conjugated poly(ε-caprolactone)-block-poly(ethylene oxide)-triphenyl phosphonium (CDDP-PCL-PEO-TPP), which was conjugated with collagenase through click chemical reaction and coated with biocompatible chondroitin sulfate by electrostatic adsorption to avoid collagenase inactivation in blood circulation. After nanoscavengers with a smaller size reached the tumor sites through enhanced permeability and retention (EPR) effect, PBAE segments changed from hydrophobic to hydrophilic due to the acidic tumor microenvironment-induced protonation of the tertiary amino group, resulting in the dissociation of partial collagenase-containing components (Fig. 3a). The collagenase-containing components served as the scavengers to promote the enzymatic digestion of collagen fibers which were abundant in tumor ECM as one of the main penetration barriers. Meanwhile, polarity change of residual collagenase-containing components in the micellar matrix furtherly caused an expansion of nanoparticles with a size increasing to 250 nm, leading to enhanced tumoral retention by preventing them from returning to the blood circulation (Fig. 3b). Subsequently, when nanoparticles entered into tumor cells by endocytosis, they could specifically target mitochondria via TPP-mediated specific recognition, making mitochondrial membrane potential to decrease and permeability to increase. The cisplatin drugs released in respond to intracellular GSH diffused into mitochondria and resulted in destruction of mitochondrial DNA, which would trigger an enhanced chemotherapeutic efficiency because of the disruption of mitochondrial repair pathway. As a consequence, these nanoscavengers could remarkably improve the antitumor effect of chemotherapy by simultaneously increasing intratumoral accumulation and precisely targeting mitochondria.

In the similar mechanisms, Tang’s group prepared a hybrid pH-sensitive alginate-based nanogel loaded with DOX and surface functionalized with collagenase for enhanced chemotherapy [46]. The modified collagenase could improve tumor-targeted drug release and penetration, and therefore deeper chemotherapy mediated by collagenase and DOX had a good inhibitory effect on the growth of H22 tumors. Du’s group constructed a “nanoenzyme capsule” by conjugating collagenase nanocapsules with heavy-chain ferritin nanocages encapsulating with DOX to enhance tumor penetration of nanoparticles through hydrolyzing collagen in tumor ECM [47]. As expected, collagen was effectively degraded by nanoenzyme capsules to increase the accumulation and penetration of nanoparticles in the solid tumor site and alleviate tumor hypoxia to enhance the antitumor efficacy of DOX for treatment of 4T1 tumors. Cao’s group recently reported collagenase IV and clusterin dual-conjugated polycaprolactone-PEG nanoparticles to load DOX for deepening the penetration of nanoparticles in dense tumors for chemotherapy [48]. Under the enzymatic hydrolysis of collagen by collagenase IV, the nanoparticles effectively aggregated in the tumors and released DOX caused by pH changes. Although more detailed study should be conducted on potential toxicity issues, these nanoparticles showed excellent antitumor effects in MCF-7 tumor-bearing nude mice.

Bromelain-based nanomedicines have also been developed for enhanced cancer chemotherapy due to the capability of bromelain to degrade tumor ECM. In this regard, Tang’s group constructed a bromelain-conjugated and lactobionic acid-modified chitosan nanoparticle as a DOX nanocarrier for cancer therapy [49]. The tumor-targeted lactobionic acid-modified chitosan nanoparticles were surface immobilized with bromelain, followed by loading with DOX. Compare to free DOX, the nanoparticles with a larger number delivery of DOX were more easily accumulated in tumor and internalized by the HepG2 cells due to the EPR effect and lactobionic acid-mediated active targeting mechanism as well as proteolysis of tumor ECM triggered by bromelain, thereby resulting in improved chemotherapeutic efficacy while reducing the side effects of cytotoxicity to normal tissues caused by anticancer drugs. In vivo experiments showed that the penetration and diffusion ability of nanoparticles in tumor area were increased upon the digestion of ECM by bromelain, leading to high drug concentration in tumor sites and superior antitumor effect. The authors similarly developed a pH-sensitive bromelain nanoparticle by ortho ester crosslinkage for enhanced DOX penetration in tumors [50]. In their study, bromelain was crosslinked by the ortho ester monomer to prepare pH-sensitive nanoparticles, which were then used to encapsulate DOX. The nanoparticles could transport more DOX into tumor sites due to the proteolysis of collagen, leading to a superior inhibition efficacy of H22 tumors.

In addition to collagen, HA is another main component in tumor ECM, and thus HAase that effectively degrades HA can allows enhanced chemotherapy. For instance, Li’s group developed a reduction/oxidation-responsive hierarchical nanoparticle with co-encapsulation of PTX and pH-stimulated HAase to achieve self-driven degradability for enhanced tumor penetration and precise chemotherapy [51]. A HA-based amphiphilic conjugate synthesized by conjugating stearic acid on HA via disulfide bonds was used to entrap PTX to form nanoparticles through self-assembly, and then pH-stimulated HAase was loaded onto the shell of nanoparticles. The nanoparticles preferentially accumulated into tumor tissues after the acid activation of pH-stimulated HAase-mediated penetration and accelerated internalization on account of the binding between HA and specific CD44 receptors. In tumor cells, the disulfide bonds were returned to sulfhydryl or oxidized to sulfoxide or sulfone under oxidation or reduction condition provided by abundant H_2_O_2_ or GSH, respectively; and pH-stimulated HAase was further activated in acidic environments. Activated HAase could promote the degradation of HA and thus destroyed the dense structure of tumor ECM, allowing deeper penetration of PTX and nanoparticles in tumors. In view of the abovementioned effects, the nanoparticles would completely disintegrate to release PTX. These self-degradable nanoparticles with dual-redox and pH responsivity showed the highest tumor inhibition ratio (93.71%) in MDA-MB-231 tumor-bearing mouse models. In another study, Sun and coworkers developed a HAase-combined multistage nanoparticle to increase solid tumor penetration and antitumor efficacy [52]. Anionic HAase was attached onto the surface of cationic epirubicin-loaded nanoparticles via electrostatic interaction. By degrading HA in tumor ECM, HAase improved the penetration of nanoparticles into solid tumors and thus enhanced the uptake by cancer cells. A rapid drug release from nanoparticles was triggered by proton sponge effect in lysosomes. Due to the deep penetration and pH-sensitive drug release, the nanoparticles showed an enhanced inhibition of growth of HepG2 tumors in living mice.

## Bioenzyme-based nanomedicines for phototherapy

Phototherapy with the unique advantages of noninvasiveness, good manipulation and accurate remote control has been extensively used for cancer treatment [53–55]. Such light-mediated treatment strategies including PTT and PDT enable suppression of tumor growth through conversion of light energy into heat and toxic ROS by agents [56, 57]. Bioenzyme-based nanomedicines can synergize with phototherapy to improve the therapeutic efficacy through a variety of synergistic mechanisms.

### Bioenzyme-based nanomedicines for PTT

Bioenzyme-based nanomedicines have been used for tumor treatment because they can effectively inhibit the growth of tumors through influencing biological activities at the molecular or cellular level. In addition, nanomedicine-mediated photothermal effect upon near-infrared (NIR) light irradiation can interact with the enzymes in different ways, such as regulation of enzyme activity and control of enzyme release, thus achieving a more effective synergistic effect in the treatment of cancer.

As the activity of bioenzymes is easily affected by temperature, NIR light-mediated photothermal effect can be used to regulate the activity of bioenzyme nanomedicine for precise cancer treatment. Cheng’s group used an ultrasmall platinum (Pt) nanoparticle to encapsulate different enzymes, including glucoamylase (GA), Gox, catalase (CAT) and proteinase K (ProK) to form enzyme-embedded Pt nanoparticles (E-Pt) [58]. The enzyme activity of E-Pt nanoparticles can be regulated through photothermal effect of Pt nanoparticles upon NIR irradiation for antitumor and antibacterial applications. In one example, Gox mediated the catalytic conversion of glucose into H_2_O_2_ and D-glucono-δ-lactone was embedded into nanoparticles to form Gox-Pt. In the absence of NIR irradiation, such Gox-Pt exhibited a similar enzyme activity as single Gox and the mix of Gox and Pt nanoparticles. However, in the presence of NIR light, the enzyme activity of Gox-Pt was obviously improved to produce much more H_2_O_2_ compared with single Gox and the mix of Gox and Pt nanoparticles. As a result, Gox-Pt irradiated by NIR light showed a more effective inhibition of breast cancer cells relative to Pt nanoparticles and single Gox.

**Fig. 4:**
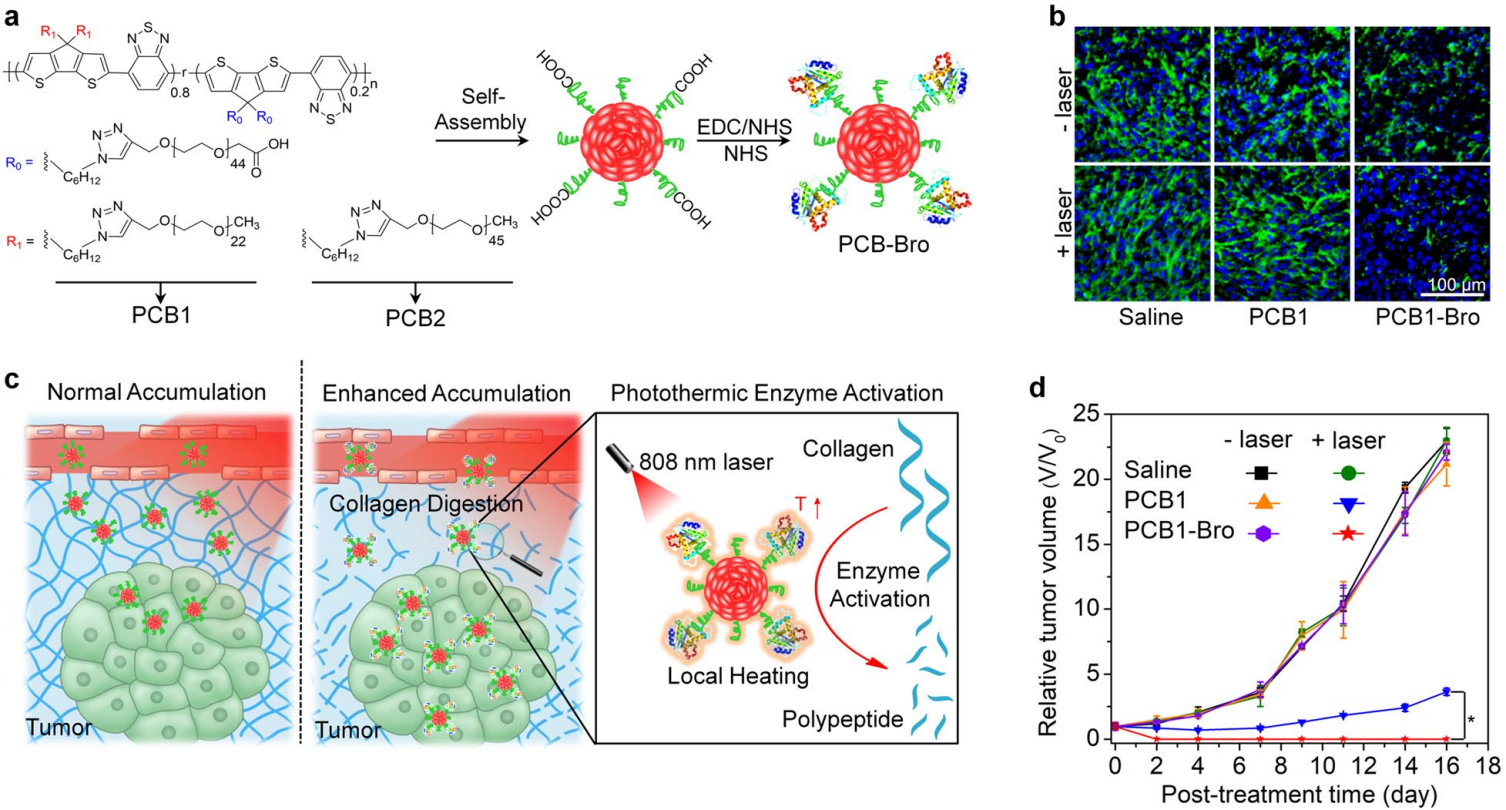
**a** Chemical structures of PCB1 and PCB2 and schematic for the synthesis of PCB-Bro. **b** Immunofluorescence collagen I staining images of 4T1 tumors after intratumoral injection of saline, PCB1, or PCB1-Bro with or without laser irradiation. Cell nucleus were stained by 4′,6-diamidino-2-phenylindole (DAPI) and collagen I was stained by Alexa Fluor 488 conjugated anti-collagen I antibody. **c** Illustration of photothermally triggered enzyme activation of PCB1-Bro towards collagen digestion for enhanced accumulation of nanoparticles in tumors. **d** Tumor growth curves of 4T1 tumor-bearing mice in different groups. Reproduced with permission from [59]. Copyright 2018, Wiley–VCH

Enzyme-mediated degradation of tumor ECM can enhance the accumulation of nanoparticles, thus amplifying PTT effect. For instance, Pu’s group reported a semiconducting polymer nanoenzyme with NIR photothermally enzymatic activity for enhanced PTT of tumors [59]. The nanoenzymes were constructed by conjugating bromelain (Bro), a temperature-responsive enzyme onto the surface of semiconducting polymer nanoparticles (SPNs) (Fig. 4a). SPNs acted as a photothermal nanotransducer to increase temperature under NIR laser irradiation. The enzymatic activity of nanoenzymes (PCB1-Bro) in the presence of NIR laser could be obviously improved, leading to degradation of collagen in tumor ECM, and more accumulation of nanoparticles in tumors (Fig. 4b,c). Thus, compared to PCB1, PCB1-Bro enabled better PTT efficacy and more efficient tumor inhibition in the 4T1 tumor-bearing mice (Fig. 4d). In addition to Bro, other enzymes have been used to synergize PTT through degrading the tumor ECM, achieving more effective tumor ablation in deeper tissues. In a recent study reported by Ping’s group, a second NIR (NIR-II) light-activated nanosystem was fabricated for precise stromal-depletion and deep-tumor PTT therapy. Such nanosystem consisted of a gold-nanorod (AuNR) core coated by mesoporous polydopamine (mPDA) and papain (Pap), a natural protease that can degrade peptides loaded inside the mPDA [60]. Due to its high efficiency of photothermal conversion (56.5%) upon NIR-II laser irradiation, such AuNR@mPDA nanoparticles were able to activate thermophilic Pap to enable on-demand enzymatic depletion of tumor ECM. AuNR@mPDA-Pap with laser irradiation was not only able to promote the release of Pap from nanosystems, but also elevated the enzymatic activity of Pap by 3.2 times relative to that without laser irradiation, thus achieving the spatiotemporal release of enzymes and on-demand stromal degradation. In HT-29 tumor-xenografted mouse model, mild NIR-II laser irradiation (0.3 W/cm^2^) for 15 min per hour was performed three times before therapeutic NIR-II laser irradiation (1.0 W/cm^2^) to trigger the release and activation of Pap and ECM degradation. As a result, the growth of HT-29 tumors treated with AuNR@mPDA-Pap and NIR-II laser irradiation was significantly inhibited, indicating amplified NIR-II PTT efficacy due to enzyme-mediated ECM depletion.

Several enzymes were able to enhance PTT efficacy by overcoming thermo-resistance of tumor cells. For example, in a recent study reported by Zhang’s group, a liquid metal nanoparticle-enzyme was developed for combinational starvation therapy and PTT of cancer [61]. The liquid metal (LM) nanoparticles were synthesized through probe sonication with the assistant of methoxy-poly(ethylene glycol) thiol (mPEG-SH), which were further coated with Gox via electrostatic interaction to obtain LM@Gox. Upon photoirradiation, LM enabled the conversion of light energy into heat efficiently to induce local hyperthermia, not only improving the Gox activity, but also accelerating blood flow and relieve the hypoxia in tumor tissues, which resulted in an improved effect of Gox-mediated starvation therapy. In addition, Gox catalysis triggered the decrease of intracellular ATP and heat shock protein (HSP) levels, which in turn overcame the severe thermo-resistance of tumor cells, eventually resulting in a superior effect of PTT. Due to the synergistic effect of enzyme treatment and PTT, LM@Gox could obviously kill 4T1 tumor cells and inhibit the growth of 4T1 tumors under photoirradiation. In another similar study, Cai’s group constructed a tumor-targeting redox-responsive composite biocatalyst to achieve the combinational action of starvation therapy and low-temperature PTT for treatment of oxygen-deprived tumors [62]. Such nanosystem was synthesized by loading Gox into porous hollow Prussian blue nanoparticles and then surface coating with HA via redox-cleavable linkage. Due to the overexpressed CD44 receptors on the surface of HepG2 cells, HA-CD44 mediated targeting pathway enabled enhanced accumulation of nanoparticles into tumors. After efficient endocytosis into tumor cells, the nanoparticles with excellent photothermal conversion efficiency and CAT-like activity could not only elevate the local temperature under photoirradiation, but also catalyzed H_2_O_2_ decomposition to produce O_2_, which contributed to enhancing the efficacy of hypoxia-suppressed Gox-mediated starvation therapy. Moreover, the Gox-mediated glucose depletion suppressed the expression levels of HSPs, which was helpful to reduce the resistance for nanoparticle-mediated low-temperature PTT, ultimately achieving more efficient enzyme-assisted PTT. Therefore, the best tumor inhibition efficacy was observed for nanoparticle treatment with NIR laser irradiation in HepG2 tumor-bearing mouse models.

Some enzymes can also be used to activate the photothermal agent prodrugs for cancer treatment. For instance, Qian’s group developed a multifunctional cascade nanoreactor with a responsive photothermal property to achieve synergistic cancer starvation therapy and PTT [63]. In their study, a photothermal agent prodrug of 3,3′,5,5′-tetramethylbenzidine (TMB), horseradish peroxidase (HRP) and Gox were loaded into hollow mesoporous silica nanoparticles (HMSN), which were encapsulated into polymer hydrogels with heat-sensitive properties to obtain an in-situ drug delivery system. Upon the accumulation of multifunctional nanoreactors into tumor sites, they underwent a phase transition and became a gel state. Once the released nanoparticles entered into tumor cells, HRP catalyzed the oxidation of TMB to activate the photothermal agents for PTT. Meanwhile, Gox-induced starvation treatment effectively inhibited the production of ATP and further inhibited the expression of HSP70, which could reduce the thermo-resistance of tumors for enhanced PTT. Such a cascade system enabled significant inhibition of the cell viability of tumor cells and ablation of solid tumors.

Nanomedicine-enabled PTT effect can also be used to control the release of enzymes to avoid the non-specific distribution of endogenous enzymes in health tissues for safe and efficient cancer therapy. For example, Yuan’s and Liu’s group reported an NIR-responsive nanoparticle to deliver exogenous enzymes for combinational chemotherapy-PTT [35]. Myrosinase (MYR), an exogenous enzyme that suppresses tumor growth by converting nontoxic glucoraphenin (GRE) into lethal sulforaphene (SFE, a kind of isothiocyanate), was encapsulated inside the aqueous cavity of liposomes, which was further coated with gold nanoparticles. NIR laser irradiation of the formed nanoparticles triggered the release of MYR. With the high accumulation of MYR at the tumor sites and photothermal effect, the enhanced catalytic efficiency could convert the nontoxic GRE into curative SFE. As such, the combinational action of chemotherapy and PTT achieved a one-time successful cure with no tumor recurrence in 4T1 tumor-bearing mice. In another study, Wu’s group constructed a thermosensitive liposome with the encapsulation of indocyanine green (ICG), Gox and gambogic acid (GA) to enhance the efficiency of mild-temperature PTT of tumors [64]. ICG-mediated PTT effect under NIR laser irradiation increased the temperature to destroy thermosensitive liposomes, leading to controlled delivery of Gox and GA at the tumor sites. Such liposomes were able to enhance the efficiency of mild-temperature PTT via synergistic inhibition of HSP expression in tumor cells caused by GA and Gox. Meanwhile, the generated H_2_O_2_ from Gox-mediated reaction would be converted into ·OH in the presence of light irradiation (400–750 nm) to effectively destruct cancer cells, achieving enzyme-enhanced PTT effect for tumors. In A549 tumor-bearing mouse models, the best antitumor efficacy was realized by enzyme-enhanced PTT. Although liposomes are excellent nanocarriers for enzymes, they usually show a low stability because of the easy oxidization and hydrolyzation of phospholipid [65], which potentially leads to unwanted leak out of enzymes from liposomes. Therefore, the stability of liposomes should be further improved to achieve controlled release of enzymes for cancer therapy.

### Bioenzyme-based nanomedicines for PDT

PDT utilizes photosensitizers to covert molecular O_2_ to cytotoxic singlet oxygen (^1^O_2_) in the presence of light at appropriate wavelength for suppression of tumor growth [66]. Various enzymes, such as CAT and Gox are able to synergize PDT through modulating the oxygen content of tumors and consuming intratumoral glucose. PDT effect can also be used to control the precise release of toxic enzymes in tumor sites for synergistic cancer therapy. Therefore, a lot of bioenzyme-based nanomedicines have been recently constructed for enhanced PDT of cancer.

CAT can efficiently reoxygenate the hypoxic tumor via converting endogenous H_2_O_2_ into O_2_ to enhance the curative effect of PDT [67, 68]. For example, Zhao’s group developed a CAT-based therapeutic nanosystem for enzyme-enhanced PDT of solid tumors [69]. In this nanosystem, β-cyclodextrin modified HA was conjugated with CAT to form HA-CAT nanoparticles, which were then loading with adamantane-modified Chlorin e6 (aCe6), leading to the formation of HA-CAT@aCe6 nanoparticles. Such HA-CAT@aCe6 nanoparticles contained three key parts: HA was a targeting molecular of CD44 receptor; aCe6 acted as a photosensitizer to produce ^1^O_2_ under laser irradiation for PDT; CAT could catalyze the endogenous H_2_O_2_ into O_2_ in situ for relieving hypoxia, improving the PDT effect. Because HA targets overexpressed CD44 receptors on cancer cells, HA-CAT@aCe6 showed a selective tumor accumulation capacity in MDA-MB-231 tumor-bearing nude mice. A higher tumor suppression ratio was achieved after intravenous injection of HA-CAT@aCe6 under 660 nm light irradiation, especially compared to the control system without CAT loading. In a similar study, Hest’s group developed a CAT loaded intelligent multifunctional synergistic nanoplatform for T_1_-weighted magnetic resonance (MR) imaging-guided enhanced PDT of HeLa tumors [70]. Lin’s group also constructed Ce6 and CAT loaded rare earth upconversion nanoparticles (UCNPs) to realize NIR light-activated catalytic-enhanced PDT [71].

Gox-mediated starvation therapy can also be used to synergize PDT. Cheng’s and Liu’s group reported a protocell-like nanoreactor (Gox-MSN@MnPc-LP) for synergistic Gox-mediated starvation therapy and PDT [72]. In the nanoreactor, the hydrophilic Gox was loaded in the pores of MSNs, while the hydrophobic manganese phthaleincyanide (MnPc) serving as a photosensitizer was loaded in the membrane layers of liposome. Gox enabled the consumption of intracellular glucose for starvation therapy and also broke the redox balance through generating H_2_O_2_, which could enhance the MnPc-mediated PDT effect under 730 nm laser irradiation. Owing to the protective effect of the nanocarriers, the monolithic structure enabled the nanoreactors to maintain the activity of enzyme for cellular delivery. Gox-MSN@MnPc-LP could possess catalysis at pH 4.0, while free Gox completely lost its function at pH 5.0. Upon intravenous administration into 4T1 tumor-bearing mice, a better therapeutic effect could be observed for the combination therapy with Gox-MSN@MnPc-LP under laser irradiation. In another study, a biomimetic hybrid nanozyme was constructed through integrating natural enzyme Gox with manganese dioxide (MnO_2_) nanozyme for enhancing the efficiency of PDT and starvation therapy [73]. The Gox and bovine serum albumin-Ce6 were conjugated on the surface of MnO_2_ nanoparticles to prepare hybrid nanozymes as the core, which were further coated by red blood cells’ membranes, leading to formation of biomimetic hybrid nanozymes. The nanozymes catalyzed endogenous H_2_O_2_ to generate O_2_ in situ, and thus provided O_2_ for Gox-mediated starvation therapy and Ce6-mediated PDT. In turn, Gox produced a large amount of H^+^ to accelerate MnO_2_-mediated O_2_ generation. Thus, the nanozymes enabled the maximization of O_2_ generation capacity of MnO_2_ and glucose consumption capacity of Gox for antitumor therapy. In the homologous 4T1 tumor-bearing mouse models, the best antitumor effect was shown in nanozymes with laser irradiation group due to the enzyme-enhanced PDT effect.

Dual-enzyme systems have also been utilized to enhance the therapeutic efficacy of PDT through intrinsic synergy effect [74]. For example, Huang’s group developed a biodegradable and O_2_ self-supplying nanoplatform for tumor microenvironment-specific activatable cascade catalytic reactions-augmented PDT [75]. Gox encapsulated manganese-doped calcium phosphate nanoparticles were co-loaded with CAT and sinoporphyrin sodium as the photosensitizer to obtain the nanoplatforms. Once internalization into 4T1 tumor cells, the endogenous H_2_O_2_ was catalyzed to generate O_2_ by CAT, which not only facilitated Gox catalytic reaction to consume more intratumoral glucose, but also alleviated tumor hypoxia and elevated the production of cytotoxic ^1^O_2_ from light-triggered photosensitizers. In 4T1 tumor-bearing mouse models, the treatment of nanoplatforms and laser irradiation with only half of the injecting dosage of other groups showed a complete inhibition of tumor growth and elimination of tumors without recurrence, suggesting the best therapeutic effect. In another similar study, a tumor-targeted cascade bioreactor with encapsulations of Gox and CAT in cancer cell membrane-camouflaged porphyrin metal–organic framework (MOF) was developed for synergistic action of enzyme-mediated starvation therapy and PDT [76]. The nanoparticles with the abilities of immune escape and homotypic targeting enabled cancer targeting and retention abilities through biomimetic surface functionalization. Once entering into tumor cells, such nanoparticles were able to elevate the oxygenation by catalyzing H_2_O_2_ to generate O_2_, which would subsequently promote the decomposition of intracellular glucose and enhance the production of PDT-mediated cytotoxic ^1^O_2_. As a result, after injection of nanoparticles with laser irradiation, the inhibition rate of 4T1 tumors was up to 97.1%, which was at least 1.1-fold higher than that in the other treatment groups.

**Fig. 5:**
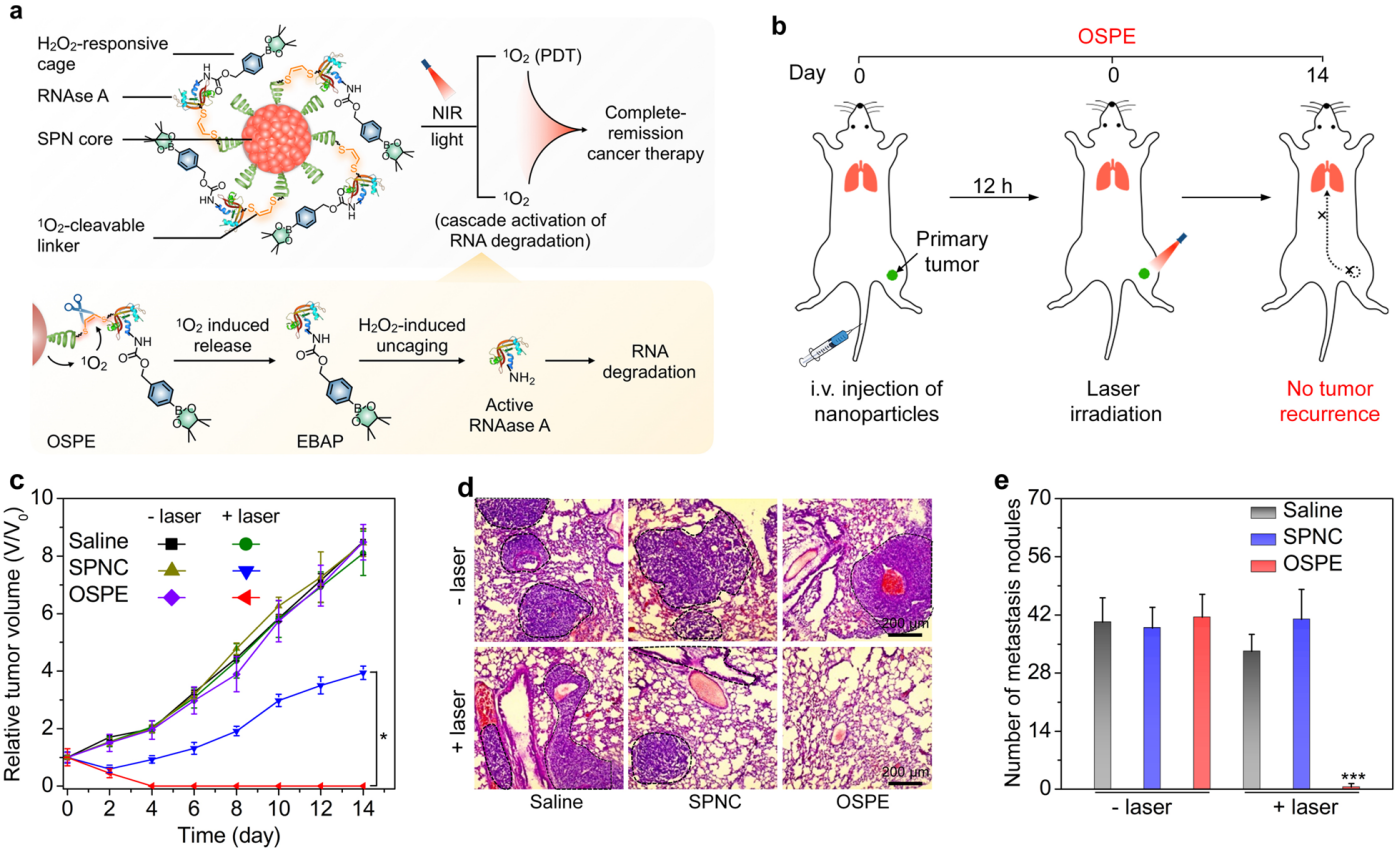
**a** Illustration of the proposed mechanism for the photoactivated synergistic therapeutic action of organic semiconducting polymer pro-nanoenzyme (OSPE) including PDT and intracellular RNA degradation. **b** Schematic illustration of OSPE-mediated complete inhibition of tumor growth and metastasis. **c** The tumor growth curves in 4T1 tumor-bearing mice. **d** Histological hematoxylin and eosin staining of pulmonary metastatic tumors in 4T1 tumor-bearing mice. Tumors were indicated by the black dashed curves. **e** Quantitative analysis of pulmonary metastatic nodules in different treatment groups. Reproduced with permission from [77]. Copyright 2019, American Chemical Society

In addition to the enhanced PDT effect by enzymes, PDT action can be used to regulate enzyme activity to obtain enhanced therapeutic efficacy. For instance, Pu’s group constructed a semiconducting polymer pro-nanoenzyme (OSPE) with a NIR photoactivatable feature for metastasis-inhibited cancer therapy [77]. Such an OSPE contained a SPN core, which was conjugated with an inactive proenzyme (EBAP) through a ^1^O_2_-cleavable linker (Fig. 5a). The EBAP was derived from cytotoxic RNase A that degrades intracellular RNA to trigger cell death, and its activity was suppressed by caging with a H_2_O_2_-responsive phenylboronic acid pinacol group. Upon irradiation of NIR laser, the SPN produced ^1^O_2_ via PDT effect, which also led to the cleavage of ^1^O_2_-responsive linker to release EBAP. The proenzymes were subsequently deprotected by intratumoral H_2_O_2_, restoring their enzyme activity for cancer-specific RNA degradation. After intravenous injection of OSPE into 4T1 tumor-bearing mouse models, followed by local 808 nm photoirradiation, the growth of primary tumors was inhibited (Fig. 5b, c). In addition, reactivated enzyme-mediated RNA degradation impeded the translation of mRNA and thus downregulated the expression of metastasis-related proteins, ultimately contributing to the complete inhibition of lung metastasis (Fig. 5d, e).

## Bioenzyme-based nanomedicines for enhanced CDT

CDT that induces cell apoptosis/necrosis by utilizing agents to convert H_2_O_2_ into hydroxyl radical (·OH) via Fenton/Fenton-like reactions is an emerging cancer treatment strategy [78]. The low concentration of endogenous H_2_O_2_ in tumors and high concentration of reducing substances (such as GSH) in the tumor microenvironment often weaken the therapeutic effect of CDT [79]. As some bioenzymes can change the levels of H_2_O_2_ and GSH, bioenzyme-based nanomedicines have been developed for enhanced CDT.

Gox can catalyze the glucose decomposition to produce H_2_O_2_ and thus increase CDT effect. As reported by Pang’s group, a multifunctional Gox-loaded nanoreactor was developed to mediate CDT, starvation therapy and sonodynamic therapy (SDT) for synergetic inhibition of 4T1 tumors [80]. Via immobilizing Gox onto a MOF and hydrogen-bonded organic framework (HOF)-based nanosystem, the nanoreactor was fabricated and could be used as Fenton’s reagent, sonosensitizer, and modulator. HOFs exhibited the potential for SDT due to the organic ligands mainly connected by hydrogen bonds. The nanoreactors could be taken up by tumor cells via endocytosis after systemic administration. Upon ultrasound irradiation, the nanoreactors induced a burst generation of ^1^O_2_ to achieve SDT. Meanwhile, the immobilized Gox could catalyze the decomposition of glucose in tumor cells to produce gluconic acid and H_2_O_2_ to realize starvation therapy. The generated H_2_O_2_ further improved the therapeutic effect of CDT through Fenton reaction. Such nanoreactors thus realized an augmented antitumor effect via synergetic CDT/SDT/starvation therapy and inhibited the growth of 4T1 tumors.

Jiang’s group reported the integration of Gox with iridium oxide (IrOx) nanoparticles to form nanozymes for synergistic treatment of breast cancer [81]. The nanozymes were constructed by loading Gox onto IrOx nanoparticles that showed intrinsic multienzyme mimetic activities similar to natural CAT, peroxidase, and oxidase. In tumor tissues, IrOx-Gox could convert the overexpressed H_2_O_2_ into O_2_, and O_2_ then reacted with continuously supplied glucose to produce H_2_O_2_ mediated by Gox, thus enabling a continuous supply of O_2_ and H_2_O_2_. As IrOx also had the activities of oxidase and peroxidase, O_2_ and H_2_O_2_ generated previously acted as substrates to deactivate IrOx. Moreover, IrOx could consume GSH through self-cyclic valence alternation of Ir^IV^ and Ir^III^ to break the antioxidation defense system of tumor cells. Such a strategy of amplified ROS generation via various ways to break the evolutionary fitness of chaotic tumors had a good inhibitory effect on tumor growth.

**Fig. 6:**
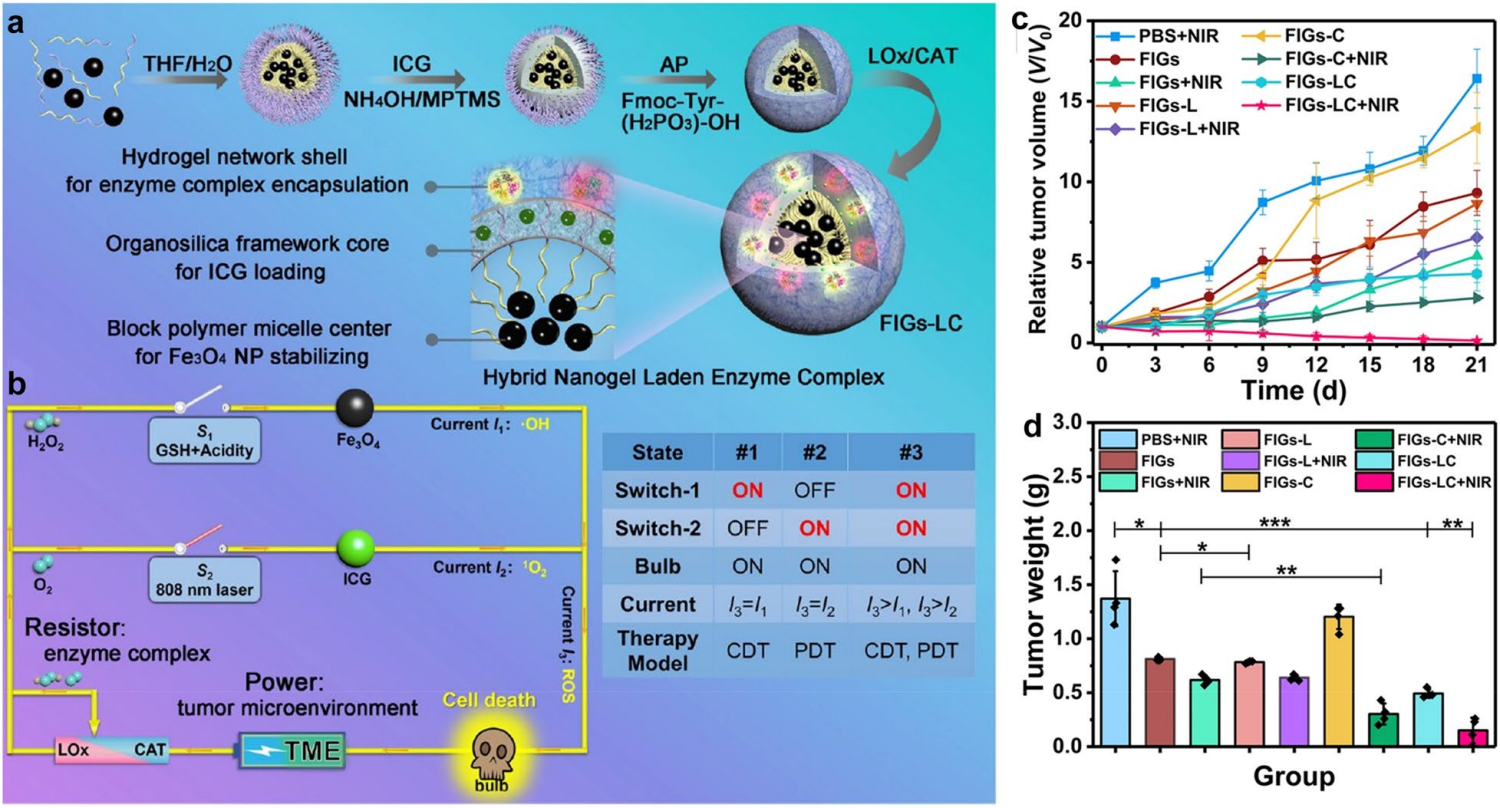
**a** The synthetic procedures of hybrid enzyme nanogel (FIGs-LC) include: (1) self-assembly of Fe_3_O_4_ nanoparticles-encapsulated polystyrene-*block*-poly (acrylic acid) (PS-*b*-PAA) micelles (Fe_3_O_4_@PS-*b*-PAA) in selective solution; (2) the formation of ICG-loaded hybrid nanoparticles (Fe_3_O_4_@IHPs) by doping ICG and introducing organic silane 3-mercaptopropyltrimethoxysilane (MPTMS); (3) acid phosphatase (AP)-triggered hydrogel coating onto Fe_3_O_4_@IHPs, denoted with Fe_3_O_4_@IHPs nanogels (FIGs); (4) immobilization of LOx and CAT into FIGs, the final particles are denoted by FIGs-LC. **b** Schematic circuit diagram for the peroxisome-inspired therapeutic mechanism of FIGs-LC based on the dual-enzyme-regulated ROS generation with GSH and NIR activation: the intratumoral lactate and H_2_O_2_ are catalyzed by LOx and CAT (resistor regulation) to generate H_2_O_2_ and O_2_, respectively. Then, H_2_O_2_ is used to produce·OH (current *I*_1_) in the presence of Fe_3_O_4_ nanoparticles within GSH-enriched acidic tumor microenvironment (Switch-1, *S*_1_), leading to the cancer cell death (bulb on). In the reaction, the produced O_2_ is converted into ^1^O_2_ (current *I*_2_) under the irradiation of an 808 nm laser (Switch-2, *S*_2_), which can also cause significant cell death (bulb on). Both CDT and PDT can be executed independently and induce the death of cancer cells, as well be activated simultaneously (*S*_1_ and *S*_2_ are connected) to achieve improved antitumor therapy. **c** Change in relative tumor volume during the treatment. **d** Average weight of excised tumors from the tumor-bearing mice after 21 days of treatment. Reproduced with permission from [82]. Copyright 2021, Springer Nature

By using lactate oxidase (LOx) and CAT as enzymes, Li’s group reported a hybrid enzyme nanogel to achieve the regulation of intracellular ROS levels for enhanced CDT and PDT of SMMC-7721 tumors [82]. They encapsulated LOx and CAT into Fe_3_O_4_/ICG co-loaded hybrid nanogels to form the hybrid enzyme nanogels (Fig. 6a). The LOx-CAT cascade system was bioinspired based on peroxisome, a special cytoplasmic organelle, which usually contained one or more enzymes, mainly oxidase, CAT, and peroxidase with H_2_O_2_ metabolism. By manipulating the ratio of LOx and CAT, the author achieved the effect of catalyzing endogenous lactic acid to produce H_2_O_2_, and further decomposing H_2_O_2_ into O_2_ by cascade (Fig. 6b). The disulfide bonds in nanogels were broken due to the higher GSH in the tumors than normal tissues, and cargos were released from the nanogels. In addition, the LOx-CAT cascade system produced H_2_O_2_ and consumed GSH in hypoxic tumors. CAT decompressed the intermediate of H_2_O_2_ and endogenous H_2_O_2_ to form O_2_. As such, ·OH was generated by Fe_3_O_4_ nanoparticles-mediated CDT and ^1^O_2_ was generated form ICG-mediated PDT under irradiation of 808 nm laser. The cascade system of LOx-CAT hybrid enzyme nanogels achieved a good therapeutic effect on SMMC-7721 tumors (Fig. 6c, d).

DNAzyme-based CAT silencing can disturb the intracellular redox microenvironment and thus improve CDT efficacy. Bu’s group constructed a DNAzyme-loaded zeolitic imidazole framework (ZIF) to disrupt redox homeostasis for treatment of hypoxic tumors [83]. They encapsulated ferrous cysteine-phosphotungstate (FeCysPW) into ZIF-82, followed by surface conjugation with PEG-NH_2_ and CAT DNAzyme (CAT Dz) to form the nanoplatform (FeCysPW@ZIF-82@CAT Dz). ZIFs was a new type of immobilization platform for biomacromolecule and was used to immobilize Dz without changing their activities. After entering into tumor cells, this nanoplatform underwent acidic degradation to form Zn ions, CAT Dz, free FeCysPW core and imidazole ligands. Zn ions assisted CAT Dz to silence CAT mRNA in tumors, resulting in excessive accumulation of H_2_O_2_. Imidazole ligands, as an electrophilic ligand, induced hypoxia-dependent GSH depletion to trigger redox dyshomeostasis. The accumulated H_2_O_2_ and FeCysPW mediated enhanced CDT to efficiently kill tumor cells. This strategy of disrupting redox homeostasis through combining CAT Dz with CDT significantly inhibited the growth of HeLa tumors in BALB/c nude mice.

## Bioenzyme-based nanomedicines for enhanced RT

RT that utilizes ionizing radiation to produce ROS can induce DNA damage, and thus can be used for cancer therapy [84]. However, the characteristics of tumor microenvironment, such as hypoxia often compromises the therapeutic efficacy of RT [85]. Recently, bioenzyme-based nanomedicines have shown the possibility to modulate the tumor microenvironment for enhanced RT.

Liu’s group reported a CAT-loaded tantalum oxide (TaOx) nanoshell for enhanced cancer RT [86]. CAT was encapsulated within hollow TaOx nanoshells and the nanoparticle surface was functionalized with PEG. The TaOx nanoparticles could be used as radiosensitizers to absorb X-ray and produce ROS for RT. The encapsulated CAT within nanoparticles decomposed H_2_O_2_ in tumor tissue to generate O_2_ and thus relieved tumor hypoxia, which greatly improved the RT effect in a mouse tumor model.

**Fig. 7:**
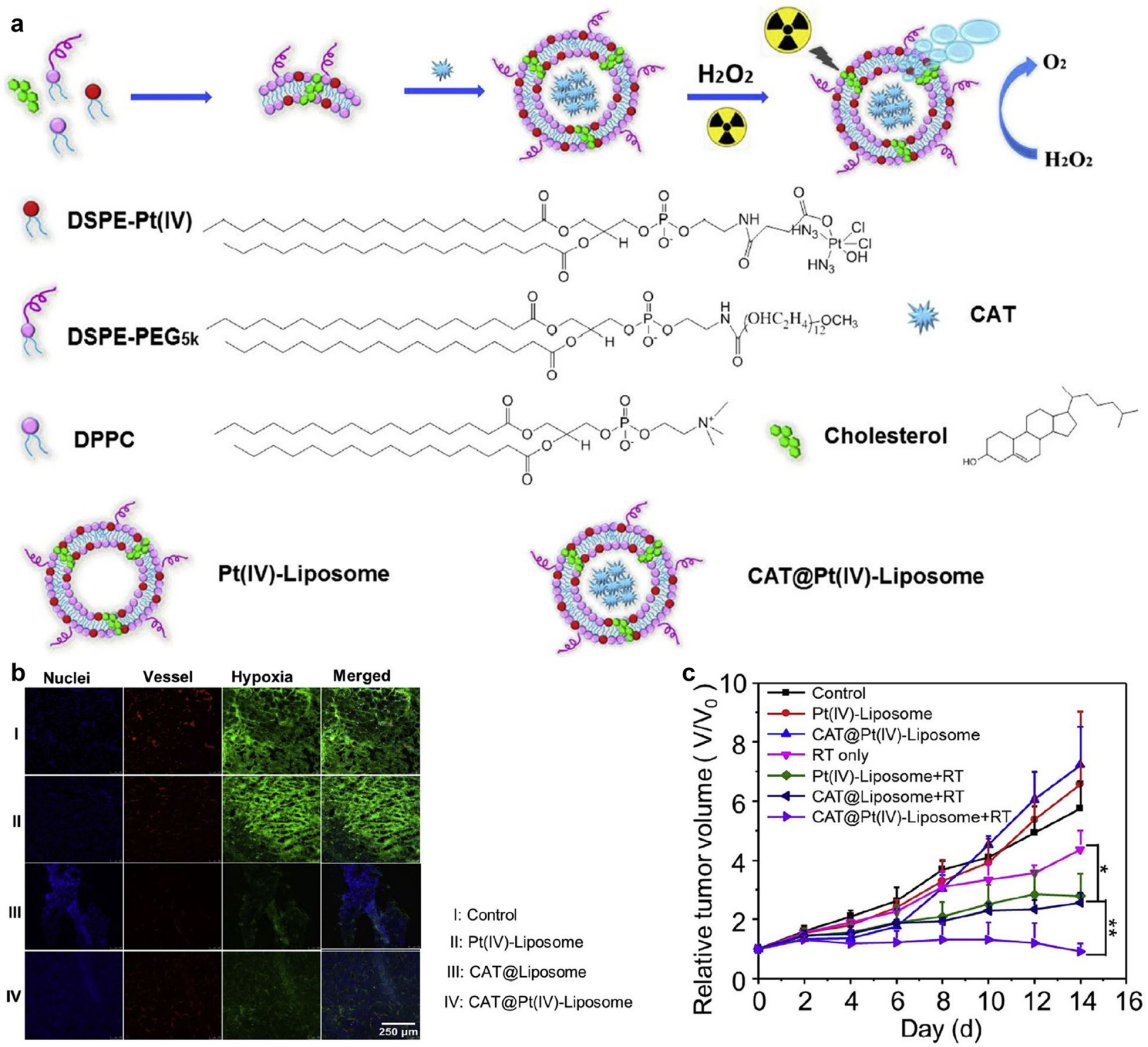
**a** A scheme showing the preparation of CAT@Pt(IV)-liposomes for tumor hypoxia relieved cancer chemo-RT. **b** Ex vivo immunofluorescence staining of tumor slices collected from mice with intravenous injection of saline, Pt(IV)-liposomes, CAT@liposomes, or CAT@Pt(IV)-liposomes at 24 h. **c** Tumor growth curves of mice after various different treatments indicated. Reproduced with permission from [87]. Copyright 2017, Elsevier

In another study, Liu’s group constructed a CAT-loaded cisplatin-prodrug-based liposome to overcome tumor hypoxia for enhanced chemo-RT of cancer [87]. CAT was encapsulated inside liposomes containing a cisplatin (IV)-prodrug-conjugated phospholipid to form CAT@Pt(IV)-liposomes (Fig. 7a). Due to CAT-mediated decomposition of H_2_O_2_, additional O_2_ was generated for relieving hypoxic tumor microenvironment. Under X-ray radiation, the highest level of DNA damage in cancer cells was observed after treatment with CAT@Pt(IV)-liposomes. Such liposomes showed an effective accumulation in tumor sites after systemic administration, and could obviously relieve hypoxic status (Fig. 7b). As a result, CAT@Pt(IV)-liposome-mediated chemotherapy and enhanced RT effectively inhibited the growth of subcutaneous 4T1 tumor in living mice (Fig. 7c).

## Bioenzyme-based nanomedicines for enhanced immunotherapy

Immunotherapy that causes the activation of systematic immunity to kill cancer cells has emerged as a promising approach for cancer treatment [88, 89]. Different from other therapeutic modalities that only be effective for local tumors, immunotherapy can be used to treat both local and metastatic tumors [90]. Moreover, immunotherapy is able to cause immune memory to prevent tumor recurrence [91]. In recently years, some bioenzyme-based nanoplatforms have been constructed for immunotherapy.

Enzymes can be used to improve the therapeutic efficacy of nanomedicines, and therefore amplify antitumor immunity. Sun’s group reported a carbon dots (CDs)-based nanoparticle to improve tumor treatment by combining starvation therapy, PDT/PTT with checkpoint-blockade immunotherapy [92]. Mn/Cu-doped CDs as photosensitizers and self-supplied oxygenators and Gox were encapsulated into polyvinyl alcohol (PVA) and poly(γ-glutamic acid) (γ-PGA)-based nanoparticles. γ-PGA coated on the surface of nanoparticles improved enhance tumor cell internalization and tumor retention through a γ-glutamyl transpeptidase-mediated endocytosis pathway. Gox could change glucose into gluconic acid and H_2_O_2_ for starvation therapy. Mn/Cu-doped CDs not only mediated PTT/PDT under 730 nm laser irradiation, but also acted as nanoreactors to induce the decomposition of H_2_O_2_ to produce O_2_ to relieve tumor hypoxia and further increase the efficiency of PDT. The nanoparticles exhibited an effective accumulation at 4T1 tumor sites after systemic administration. After irradiation with NIR light, tumor cells were killed by enhanced PDT, PTT and starvation therapy. After the combination with checkpoint blockade immunotherapy with PD-L1 antibody, the promoted PDT therapy would effectively enhance the infiltration of cytotoxic T lymphocytes into distant tumors. As such, the nanoparticles achieved a good inhibitory effect on 4T1 primary and distant tumors.

To overcome the challenge of tumor hypoxia and achieve ideal photodynamic systemic antitumor immunity, Liu’s group similarly designed a unique type of hollow silica smart nanoreactor with the property of pH-responsive charge-convert, mitochondria-targeting and PDT effect [93]. To obtain the nanoreactor, hollow silica nanoparticles with H_2_O_2_-decomposing enzyme (CAT) encapsulated within inner cavities and photosensitizer Ce6 doped into the silica lattice structures were modified with (3-carboxypropyl)triphenylphosphonium bromide (CTPP), a mitochondria targeting molecule and a pH-responsive polymer, PEG/2,3-dimethylmaleic anhydride (DMMA) co-grafted poly(allylamine hydrochloride) (PAH) via electrostatic interactions. Once within the acidic tumor microenvironment after intravenous injection, the nanoreactor would exhibit enhanced binding and uptake by tumor cells and more efficient retention in tumor tissues because of pH-responsive charge conversion and escape endosome/lysosome of “proton-sponge” effect. After pH-responsive de-shielding of polymer coating, the superficially modified CTPP was exposed to mediate effective intracellular mitochondrial targeting. Meanwhile, CAT encapsulated in nanoparticles decomposed H_2_O_2_ into oxygen for overcoming tumor hypoxia and further enhancing PDT efficacy. By combination with check-point blockade immunotherapy via additionally intravenous injection of programmed death-ligand 1 (PD-L1) antibody, the enhanced PDT treatment could significantly promote the infiltration of cytotoxic T lymphocytes (CTLs) into distant tumors and inhibit the growth, demonstrating a strong abscopal effect in metastasis inhibition. Such a strategy was able to simultaneously address several limitations of conventional PDT and might be promising for treating both local tumors and distant tumor metastases.

In addition to PDT, enzymes have been used to enhance RT-combined immunotherapy. As an example, Liu’s group utilized core/shell poly(lactic-co-glycolic) acid (PLGA) nanoparticles to encapsulate CAT and imiquimod (R837), a Toll like receptor-7 agonist, as an immune adjuvant for RT-combined immunotherapy [94]. CAT triggered rapidly decomposed of H_2_O_2_ to produce O_2_, and thus the hypoxia status in tumor microenvironment was improved. This method could greatly increase the effectiveness of RT, which is highly dependent on the concentration of O_2_. After an amplified RT using PLGA-R837@CAT to generate more tumor debris, a more powerful antitumor immune response was caused by the released R837. The core–shell nanoparticles were then combined with a checkpoint blockade antibody to achieve a systemic synergistic therapeutic effect, resulting in inhibition of primary, metastatic, and recurrent tumors in CT26 tumor mouse models.

Besides relieving tumor hypoxia, enzyme can enable ECM degradation and enhanced penetration of nanoparticles to amplify the efficacy of immunotherapy. Liu’s group developed a HAase-modified pH-responsive nanoparticle to achieve PDT-combined immunotherapy on bilateral CT26 tumor model [95]. HAase was conjugated with dextran (DEX) via a pH-responsive linker to form the nanoparticles (DEX-HAase). In acidic tumor microenvironment, DEX-HAase would be dissociated to release native HAase, which induced ECM degradation and subsequently led to enhanced penetration of O_2_ and other therapeutic agents. The largely relieved tumor hypoxia promoted PDT effect of Ce6@liposomes, and reversed the immunosuppressive tumor microenvironment to boost antitumor immunity. As such, the therapeutic effect achieved by the combination of PDT and anti-PD-L1 checkpoint blockade therapy could be significantly enhanced by pretreatment with DEX-HAase. Such treatment could destruct both primary tumors with direct PDT effect, and distant tumors via a robust abscopal effect.

Enzyme-mediated ECM degradation can facilitate the infiltration of effector T cells into tumors to improve the efficacy of immunotherapy [96]. Shen’s group reported a microneedle to load HAase-conjugated SPNs and an immune adjuvant polyinosinic-polycytidylic acid (PIC) for PTT and immunotherapy of melanoma [97]. After piercing at tumor sites, it could achieve delivery of HAase-modified SPNs and PIC due to the degradation of microneedles. HAase-modified SPNs dissolved the ECM to allow deep penetration of SPNs and PIC in tumor tissues. Under 808 nm laser irradiation, the microneedles mediated PTT and immunotherapy, leading to activation of immune cells and enhanced T-cell immune response for inhibition of tumor growth and metastasis of melanoma.

**Fig. 8:**
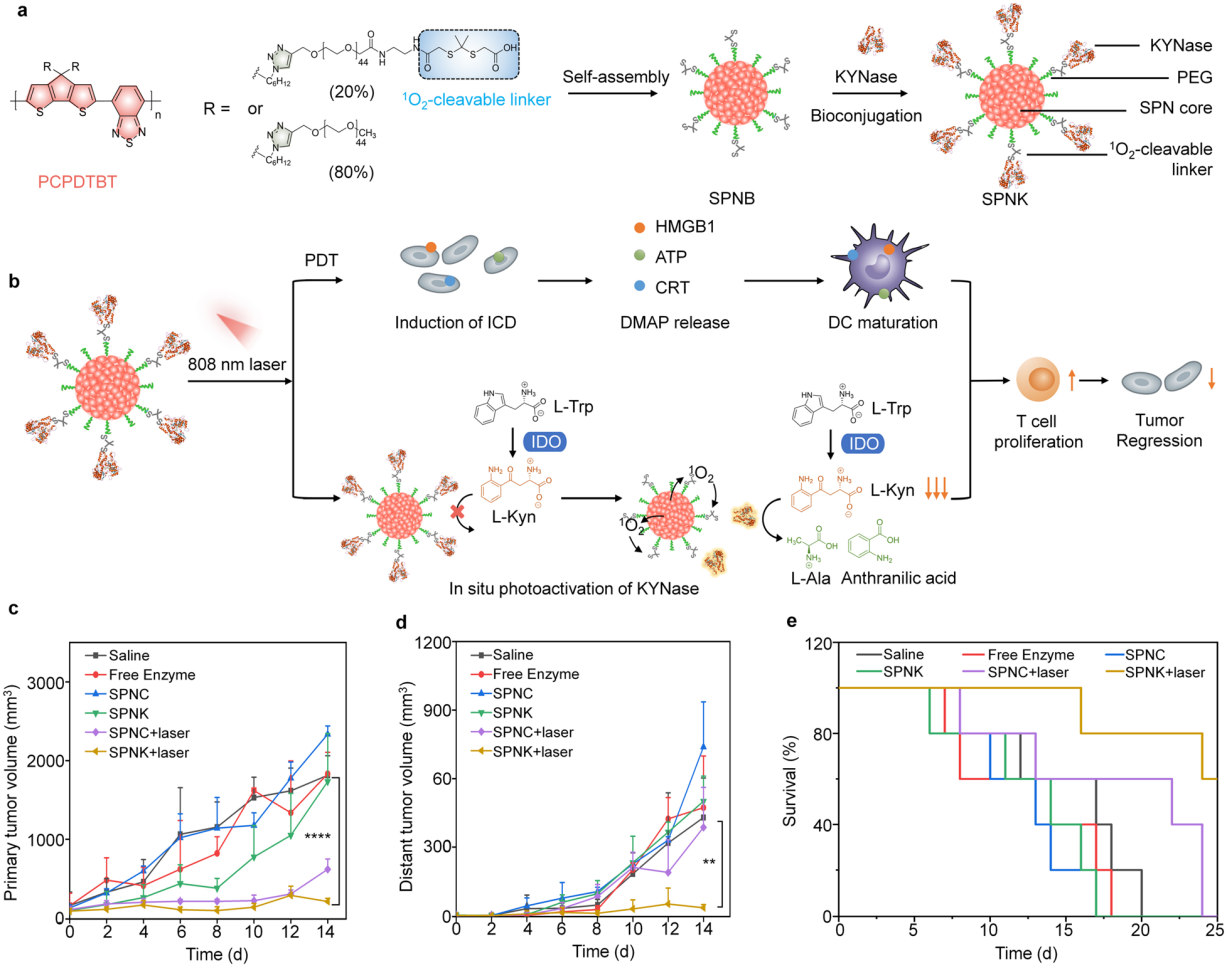
**a** Schematic illustration of the preparation procedure of SPNK. **b** The proposed mechanism of SPNK-mediated synergistic photodynamic immunometabolic therapy. Growth curves of **c** primary tumors and **d** distant tumors in B16F10 tumor-bearing mice after different treatments. **e** Survival curves for the treated and control mice. Reproduced with permission from [99]. Copyright 2021, Wiley–VCH

Kynureninase (KYNase) is an enzyme that can degrade the immunosuppressive kynurenine (Kyn) produced from tryptophan (Trp) catabolism mediated by indoleamine 2,3-dioxygenase (IDO), and thus reverses immunosuppressive tumor microenvironment [98]. Pu’s group recently constructed an activatable polymer nanoenzyme by combining KYNase-based immunometabolic therapy with PDT to treat tumors [99]. They first modified PEG with a ^1^O_2_-cleavable linker, 2,2′-(propane-2,2-diylbis(sulfanediyl) diacetic acid (PSDA) to synthesize PEG-PSDA. Then, the SPN core synthesized beforehand was wrapped with PEG-PSDA to build an intermediate product SPNB, and KYNase was conjugated onto the surface of SPNB to form an activatable polymer nanoenzyme named SPNK (Fig. 8a). The nanoenzyme could achieve controlled release of KYNase upon laser irradiation due to SPN-enabled ^1^O_2_ generation. Owing to the EPR effect after systematic administration, SPNK showed an effective accumulation in breast tumor sites. When the tumors were irradiated under an 808 nm laser, tumor cells were killed and immunogenic cell death (ICD) was induced by the generated ^1^O_2_ during PDT. The generated ^1^O_2_ could also break the ^1^O_2_-cleavable linker in SPNK to release KYNase, leading to degradation of Kyn. As the depletion of Kyn, the proliferation and infiltration of effector T cells were promoted, and thus the systemic antitumor immunity was boosted (Fig. 8b). The synergistic effect of PDT and immunometabolic therapy could effectively treat the primary and metastatic tumors in 4T1 tumor mouse models (Fig. 8c–e).

## Conclusion and perspectives

Bioenzymes have been demonstrated to have great potentials for cancer treatments. This review mainly describes the recent development of bioenzyme-based nanomedicines for cancer therapy with the emphasis on enhanced therapeutic efficacies mediated by bioenzymes (Table 1). Through directly inducing cancer cell death, modulating tumor microenvironment (such as pH, ROS, GSH level, hypoxia, ATP level, glucose concentration, HSP expression, and Kyn level), and degrading tumor ECM, bioenzyme-based nanomedicines can combine/synergize with chemotherapy, PTT, PDT, CDT, RT, and immunotherapy, resulting in significantly enhanced efficacy in treating solid tumors.

**Table 1:** Summary of bioenzyme-based nanomedicines for enhanced cancer therapy

Therapeutic modality	Nanosystem	Enzyme	Work mechanism	Reference
Chemotherapy	Nanogels	Ribonuclease A	Catalyze RNA degradation	[36]
	Mesoporous silica	Ribonuclease A	Catalyze RNA degradation	[37]
	Large-pore mesoporous silica	Ribonuclease A	Degrade mRNA and tRNA	[38]
	Polymer-based nanoparticles	Ribonuclease A	Degrade cellular RNA	[39]
	Hollow organosilica	Glucose oxidase	Consume glucose	[40]
	Mesoporous polydopamine	Glucose oxidase	Consume glucose	[41]
	Liposomes	Glucose oxidase	Consume glucose	[42]
	Porous silica nanoparticles	Hyaluronidase	Decompose HA-DOX to produce toxic dissociative DOX	[43]
	Micelles	Collagenase	Digest collagen fibers in tumor ECM	[45]
	Nanogels	Collagenase	Digest tumor ECM	[46]
	Heavy-chain ferritin nanocages	Collagenase	Degrade the collagen in tumor ECM	[47]
	PCL-PEG nanoparticles	Collagenase IV	Degrade the collagen component of ECM	[48]
	Chitosan nanoparticles	Bromelain	Digest tumor ECM	[49]
	Hierarchical nanoparticles	Hyaluronidase	Degrade hyaluronic acid in tumor ECM	[51]
	Micelles	Hyaluronidase	Degrade hyaluronic acid in tumor ECM	[52]
PTT	Ultrasmall platinum nanoparticles	Glucose oxidase	Catalyze glucose to produce H_2_O_2_ and D-glucono-δ-lactone	[58]
	Semiconducting polymer nanoparticles	Bromelain	Digest collagen in tumor ECM	[59]
	Gold/mesoporous polydopamine nanoparticles	Papain	Degrade tumor ECM	[60]
	Liquid metal nanoparticles	Glucose oxidase	Inhibit ATP and HSP levels	[61]
	Porous hollow Prussian blue nanoparticles	Glucose oxidase	Enhance glucose depletion	[62]
	Hollow mesoporous silica	Glucose oxidase	Reduce the thermo-resistance and consume glucose	[63]
	Gold nanoparticles	Myrosinase	convert nontoxic GRE into toxic SFE	[35]
	Liposomes	Glucose oxidase	Consume glucose and inhibit HSP expression	[64]
PDT	Hyaluronic-acid-based nanoparticles	Catalase	Decompose endogenous H_2_O_2_ to generate O_2_	[69]
	Polymeric micelles	Catalase	Catalyze endogenous H_2_O_2_ to generate O_2_	[70]
	Upconversion nanoparticles	Catalase	Catalyze endogenous H_2_O_2_ to generate O_2_	[71]
	Mesoporous silica	Glucose oxidase	Catalyze glucose consumption to generates H_2_O_2_	[72]
	Biomimetic hybrid nanozymes	Glucose oxidase	Oxidize glucose to gluconic acid and H_2_O_2_	[73]
	Manganese-doped calcium phosphate nanoparticles	Catalase and glucose oxidase	Convert intracellular H_2_O_2_ to O_2_ and intratumoral glucose to H_2_O_2_ and gluconic acid	[75]
	Metal–organic frameworks	Catalase and glucose oxidase	Convert intracellular H_2_O_2_ to O_2_ and consume glucose	[76]
	Semiconducting polymer nanoparticles	Ribonuclease A	Degrade intracellular RNA	[77]
CDT	Metal organic frameworks	Glucose oxidase	Catalyze glucose oxidation to produce H_2_O_2_ and gluconic acid	[80]
	Iridium oxide nanoparticles	Glucose oxidase	Oxidize glucose to gluconic acid and H_2_O_2_	[81]
	Nanogels	Lactate oxidase and catalase	Catalyze lactate to produce H_2_O_2_ and decompose H_2_O_2_ into O_2_	[82]
	Zeolitic imidazole framework	Catalase and DNAzyme	Silence CAT and deplete GSH	[83]
RT	TaOx nanoparticles Cisplatin-prodrug-based liposomes	Catalase Catalase	Degrade H_2_O_2_ to generate O_2_ Degrade H_2_O_2_ to generate O_2_	[86] [87]
Immunotherapy	γ-PGA nanoparticles	Glucose oxidase	Deplete glucose	[92]
	Tetraethyl orthosilicate	Catalase	Degrade H_2_O_2_ to generate O_2_	[93]
	PLGA nanoparticles	Catalase	Degrade H_2_O_2_ to generate O_2_	[94]
	Dextran-conjugated nanoparticles	Hyaluronidase	Degrade hyaluronic acid in tumor CEM	[95]
	Microneedles	Hyaluronidase	Degrade hyaluronic acid in tumor CEM	[97]
	Semiconducting polymer nanoparticles	Kynureninase	Degrade the immunosuppressive kynurenine	[99]

Although these recent promising progresses of bioenzyme-based nanomedicines in the field of cancer therapy have been achieved, there are still several issues remained to be addressed before their future clinical translation. First, almost all of the bioenzymes discussed in this review are not approved by the U.S. Food and Drug Administration (FDA) [100, 101]. Great efforts should be made to evaluate the properties of enzymes including immunogenicity, catalytic activity, and clearance from the body [102]. Second, uncontrolled distribution of bioactive enzymes may induce side effects to normal tissues. Approaches to regulate the activities of enzymes through conditional protein splicing [103], reversible chemical engineering [104], and photocleavable group caging [105] can achieve targeting of enzymes to specific cell and tissue sites. Alternatively, development of responsive nanocarriers to control release of enzymes in targeting sites can be adopted [106]. Third, as the activities of enzymes are often affected by surrounding environments, such as low pH and high temperature, the fabrication processes of nanomedicines may compromise the bioactivity of enzymes. In this regard, some simple and mild approaches should be adopted to fabricate bioenzyme-based nanomedicines. Fourth, long-term biocompatibility, biodegradability, and clearance of nanomedicines in living body need to be ensured. It is highly encouraged to construct nanomedicines with fully biocompatible and biodegradable materials to integrate enzymes [107]. New manufacturing methods to endow nanoparticles with good biodegradability or reduce the sizes of nanoparticles below 5 nm are also important to ensure their rapid clearance [108–110]. Fifth, due to the complicated and tedious steps to incorporate various components in bioenzyme-based nanomedicines, their large-scale manufacturing is a big challenge. Much deeper researches on new material platforms, and straightforward and universal manufacturing methods are required to address this concern. Thus, with the addressing of these great challenges, bioenzyme-based nanomedicines should be translated into clinical usage for cancer therapy.

## Data Availability

The review is based on the published data and sources of data upon which conclusions have been drawn can be found in the reference list.

## Abbreviations

ROS: Reactive oxygen species
RNase A: Ribonuclease A
Azo: Azobenzene
β-CD: β-Cyclodextrin
PLG-g-mPEG: Poly (L-glutamic acid)-graft-poly (ethylene glycol) methyl ether
CA4: Combretastatin A4
NTR: Nitroreductase
MSN: Mesoporous silica nanoparticle
GSH: Glutathione
DSP: Cis-platinum pro-drugs
DOX: Doxorubicin
RNBC: RNase A modified with a nitrophenyl tetramethyl-dioxaborolanyl benzyl carbamate group
Gox: Glucose oxidase
TPZ: Tirapazamine
ATP: Adenosine triphosphate
P-gp: P-glycoprotein
PTX: Paclitaxel
HAase: Hyaluronidase
PE: Polyester
HA: Hyaluronic acid
ECM: Extracellular matrix
MAL-PEG-PBAE: Maleimide-terminated poly(ethylene glycol)-block-poly(β-amino ester)
CDDP: Succinic anhydride-modified cisplatin
PCL: Poly(ε-caprolactone)
PEO: Poly(ethylene oxide)
TPP: Triphenyl phosphonium
EPR: Enhanced permeability and retention
RT: Radiotherapy
PTT: Photothermal therapy
PDT: Photodynamic therapy
NIR: Near-infrared
Pt: Platinum
GA: Glucoamylase
CAT: Catalase
ProK: Proteinase K
Bro: Bromelain
SPNs: Semiconducting polymer nanoparticles
NIR-II: Second NIR
AuNR: Gold-nanorod
mPDA: Mesoporous polydopamine
Pap: Papain
DAPI: 4′,6-Diamidino-2-phenylindole
LM: Liquid metal
mPEG-SH: Methoxy-poly(ethylene glycol) thiol
HSP: Heat shock protein
TMB: 3,3′,5,5′-Tetramethylbenzidine
HRP: Horseradish peroxidase
HMSN: Hollow mesoporous silica nanoparticles
MYR: Myrosinase
GRE: Glucoraphanin
SFE: Sulforaphane
ICG: Indocyanine green
GA: Gambogic acid
^1^O_2_: Singlet oxygen
aCe6: Adamantane-modified Chlorin e6
MR: Magnetic resonance
UCNPs: Upconversion nanoparticles
MnPc: Manganese phthaleincyanide
MnO_2_: Manganese dioxide
MOF: Metal–organic framework
RNA: Ribonucleic acid
OSPE: Organic semiconducting polymer pro-nanoenzyme
CDT: Chemodynamic therapy
OH: Hydroxyl radical
SDT: Sonodynamic therapy
HOFs: Hydrogen-bonded organic frameworks
IrOx: Iridium oxide
LOx: Lactate oxidase
PS-*b*-PAA: Polystyrene-*block*-poly (acrylic acid)
IHPs: ICG-loaded hybrid nanoparticles
MPTMS: 3-Mercaptopropyltrimethoxysilane
AP: Phosphatase
ZIF: Zeolitic imidazole framework
CDs: Carbon dots
TaOx: Tantalum oxide
Pt(IV): Cisplatin (IV)
PVA: Polyvinyl alcohol
γ-PGA: Poly(γ-glutamic acid)
CTPP: (3-Carboxypropyl)triphenylphosphonium bromide
DMMA: 2,3-Dimethylmaleic anhydride
PAH: Poly(allylamine hydrochloride)
PD-L1: Programmed death-ligand 1
CTLs: Cytotoxic T lymphocytes
PLGA: Poly(lactic-co-glycolic) acid
DEX: Dextran
PIC: Polyinosinic-polycytidylic acid
KYNase: Kynureninase
Kyn: Kynurenine
TrP: Tryptophan
IDO: Indoleamine 2,3-dioxygenase
PSDA: Propane-2,2-diylbis(sulfanediyl) diacetic acid
ICD: Immunogenic cell death
FDA: Food and Drug Administration
